# Supplementary material for: Targeting Mitochondria with ClpP Agonists as a Novel Therapeutic Opportunity in Breast Cancer
Source: Cancers (Basel). 2023 Mar 23;15(7):1936. doi: 10.3390/cancers15071936 (PMC10093243; doi:10.3390/cancers15071936)
Supplement: Supplementary file 1 [file cancers-15-01936-s001.zip › cancers-2222725-supplementary.pdf]

Supplementary materials

Targeting mitochondria with ClpP agonist as novel therapeutic opportunity in breast cancer

Wedam et al.

Contents

- Supplementary Figure S1: Mitochondrial metabolic pathways dysregulated in breast cancers
- Extended discussion: Status of non-ClpP mitochondria targeting therapies in breast cancers
- Supplementary Table S1: The status of development of mitochondria-targeting drugs in breast cancers (not including ClpP agonists)

Supplementary Figure S1

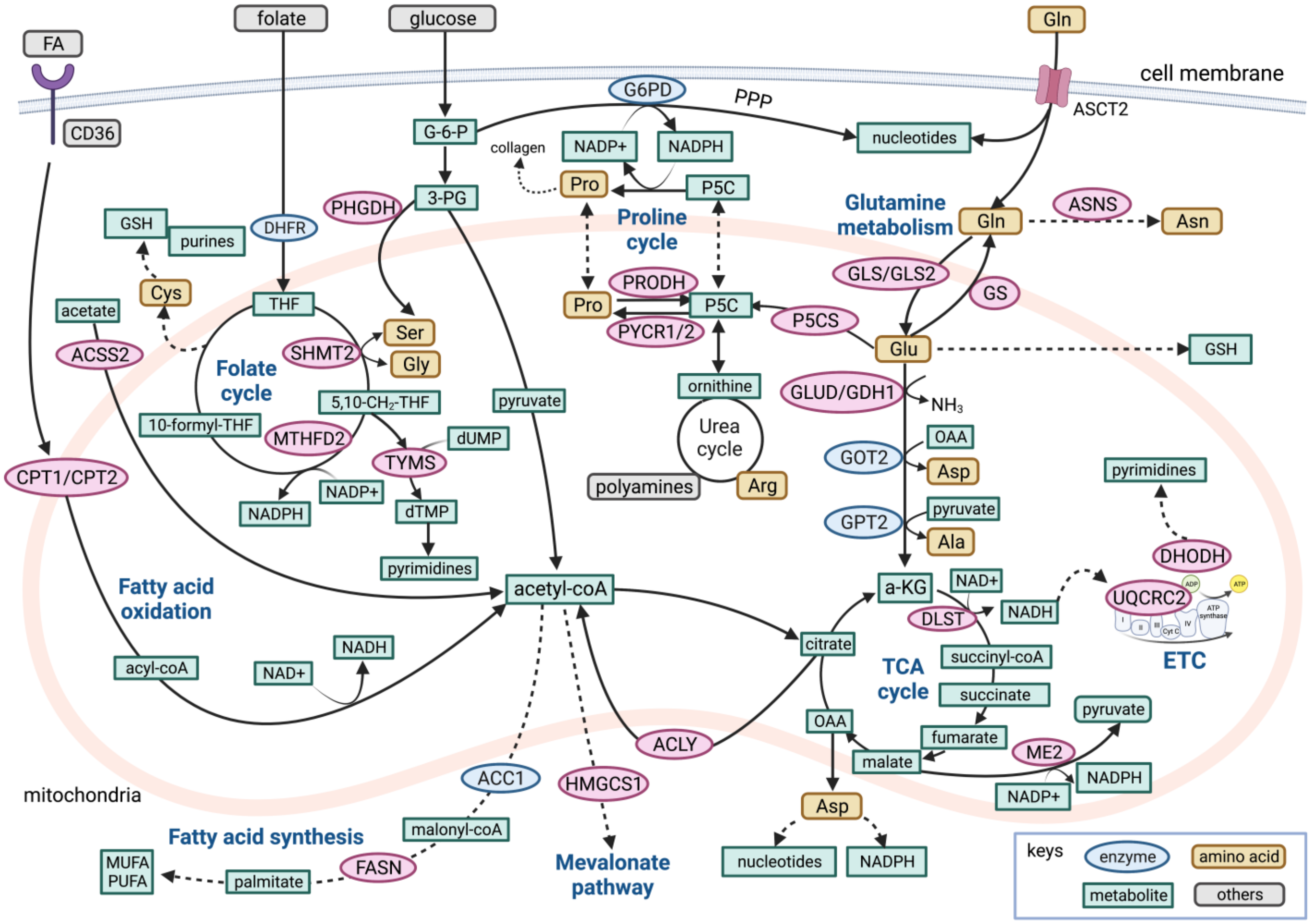

Figure S1. Mitochondrial metabolic pathways dysregulated in breast cancers. Enzymes elevated in breast cancers are highlighted with magenta. Abbreviation of enzymes. ACC1: acetyl-coA carboxylase 1; ACLY: ATP-citrate synthase; ACSS2: acetyl-coenzyme A synthetase; ASNS: asparagine synthetase; CPT1: carnitine O-palmitoyltransferase 1; CPT2: carnitine O-palmitoyltransferase 2; DHFR: dihydrofolate reductase; DHODH: dihydroorotate dehydrogenase (quinone), mitochondrial; DLST/OGDC: dihydrolipoyllysine-residue succinyltransferase component of 2-oxoglutarate dehydrogenase complex, mitochondrial/ 2-oxoglutarate dehydrogenase complex; FASN: fatty acid synthase; G6PD: glucose-6-phosphate 1-dehydrogenase;GLS: glutaminase kidney isoform, mitochondrial; GLS2: glutaminase liver isoform, mitochondrial; GLUD/GDH1: glutamate dehydrogenase 1, mitochondrial; GOT2: aspartate aminotransferase, mitochondrial; GPT2: alanine aminotransferase 2; GS: glutamine synthetase; HMGCS1: hydroxymethylglutaryl-CoA synthase, cytoplasmic; ME2: NAD-dependent malic enzyme, mitochondrial; P5CS: delta-1-pyrroline-5-carboxylate synthase; PYCR1: pyrroline-5-carboxylate reductase 1, mitochondrial; PYCR2: pyrroline-5-carboxylate reductase 2; PRODH: proline dehydrogenase 1, mitochondrial; PHGDH: D-3-phosphoglycerate dehydrogenase; SHMT2: serine hydroxymethyltransferase, mitochondrial; MTHFD2: bifunctional methylenetetrahydrofolate dehydrogenase/cyclohydrolase, mitochondrial; TYMS: thymidylate synthase. **Amino acids.** Ala: alanine; Arg: arginine; Asn: asparagine; Asp: aspartic acid; Cys: cysteine; Gln: glutamine; Glu: glutamate; Gly: glycine; Pro: proline; Ser: serine. **Metabolites and Others.** ASCT2: sodium-dependent neutral amino acid transporter type 2; a-KG: alpha-ketoglutarate; dTMP: deoxythymidine monophosphate; dUTP: deoxyuridine triphosphate; G-6-P: glucose-6-phosphate; FA: fatty acid; GSH: glutathione; MUFA: monounsaturated fatty acids; NAD+/NADH: nicotinamide adenine dinucleotide; NADP+/NADPH: nicotinamide adenine dinucleotide phosphate; OAA: oxaloacetate; P5C: delta-pyrroline-5-carboxylate; PPP: pentose phosphate pathway; PUFA: polyunsaturated fatty acids; 3-PG: 3-phosphoglyceric acid; THF: tetrahydrofolate; 5, 10-CH_2-THF: 5,10-methylenetetrahydrofolate; 10-formyl-THF: 10-formyltetrahydrofolate, UQCRC2: cytochrome b-c1 complex subunit 2, mitochondrial. The figure was generated by BioRender.com (Jan.20, 2023).

Extended discussion

1. Status of non-ClpP mitochondria targeting therapies in breast cancers

In addition to ClpP agonists, multiple other mitochondria-targeting drugs have been tested in preclinical breast cancer models and clinical trials (see Table S1 and reviews [1-3]).

1.1. ETC complex inhibitors

Tri-Phenyl-Phosphonium cation (TPP+)-based mitochondrially targeted drugs have been actively exploited in preclinical breast cancer models. Several TPP+ conjugated drugs have been developed [4-8], and among them, Tamoxifen-TPP+ (**mitoTam**), a Complex I inhibitor, is cytotoxic to ER positive, HER2-positive, TNBC, as well as tamoxifen-resistant cell lines without causing systemic toxicity [9]. MitoTam has been further tested in a phase 1 trial (EudraCT 2017-004441-25) as of 2020 [3], however, clinical results are not yet available. Other ETC inhibitors have been tested in preclinical models of breast cancer, including Complex II inhibitors, such as the vitamin E derivative (+) α-tocopheryl succinate **(α -TOS)**, and mitochondrially targeted vitamin E succinate (**MitoVES**) [133]. **IACS-010759**, a Complex I inhibitor, suppressed tumor growth in TNBC patient-derived xenografts models, and combination with palbociclib, a CDK4/6 inhibitor or the multi kinase inhibitor cabozantinib showed improved anti-tumor efficacy[10]. IACS-010759 has not yet been tested in clinical trials in breast cancers. A recent phase 1 trial in AML and solid tumors revealed neurotoxicity due to IACS-010759 and only showed modest efficacy[11]. **ME-344,** an isoflavone molecule shown to inhibit the ETC Complexes I-V [12], has shown significant antitumor activity in HER2-negative breast cancer, particularly in combination with the vascular endothelial growth factor (VEGF) inhibitor bevacizumab which induces vascular normalization and tissue reoxygenation [13]. **Metformin**, a drug widely used for type-2 diabetes, functions as an complex I inhibitor [14], although the inhibitory effect is seen at very high concentrations (IC50~20 mM) compared with that of the canonical complex I inhibitors rotenone and piericidin A (IC50~2 μM)[15]. Nevertheless, since the anti-tumor effect of metformin was reported [16], metformin has been actively tested in cancers. Anti-tumor effects of metformin in breast cancer models have been demonstrated in multiple studies, such as breast CSCs [17,18], tumor growth [18,19], lung metastasis, and chemo sensitization [20]. Despite these positive preclinical findings, clinical trials with metformin in breast cancers have had disappointing results [21-24]. Importantly, the mechanism of anti-tumor effect of metformin still remains unclear. It has been indicated that not only Complex I inhibition, but reduced availability of insulin and inhibition of mTOR signaling are potential mechanisms of the observed anti-tumor effect [25,26].

Leflunomide, a DHODH inhibitor, has been shown to effective for treatment of some cancers including breast cancer [27], and has been currently tested in clinical trial.

1.2. TCA cycle, glutamine metabolism, fatty acid synthesis, FAO inhibitors

CPI-613/Devimistat is an inhibitor of two TCA cycle enzymes, pyruvate dehydrogenase (PDH) and α-ketoglutarate dehydrogenase (a-KGDH). CPI-613 is cytotoxic to breast cancer cells [28,29]. In a phase 1 study of CPI-613 combined with FOLFIRINOX showed that safety and tolerability in patients with metastatic pancreatic cancer [30], however, two phase 3 trials failed to demonstrate enhanced efficacy in pancreatic cancer [31] and AML (NCT03504410, trial terminated). CPI-613 has not entered clinical trials for patients with breast cancers. **CB-839**, a glutaminase inhibitor, displayed significant anti-tumor activity in breast cancer models, both as a single agent and in combination with paclitaxel [32]. CB-839 is currently being tested in TNBC patients in a phase 2 clinical trial in combination with Paclitaxel (NCT03057600). **TVB-2640**, a FASN inhibitor, is currently being tested in a phase 2 clinical trial with HER2-positive breast cancer patients [33](NCT03179904). **Bempedoic acid** (BA) is an ACLY inhibitor FDA-approved for hypercholesterolemia. BA is shown to impair breast cancer growth and invasion in combination with CDK4/6 inhibitor [34]. **Omeprazole**, a proton pump inhibitor, has been shown to selectively inhibit FASN activity and induces apoptosis in TNBC cell lines [35]. Omeprazole was recently tested in a single arm phase 2 neoadjuvant clinical trial in TNBC patients in combination with chemotherapy and a pathologic complete response (pCR) of >70% was observed [36]. **Etomoxir**, CPT1 inhibitor, showed anti-tumor activity in pre-clinical breast cancer studies [37,38], but has not yet been tested in clinical trials.

1.3. Anti-apoptotic Bcl-2 family protein inhibitors

Navitoclax/ABT-263, an inhibitor of Bcl-2/Bcl-xL/Bcl-w has been tested in preclinical breast cancer models [39-45]. Navitoclax showed a limited efficacy due to high expression of Mcl-1, while combination of Navitoclax and an Mcl-1 inhibitor had a synergistic effect [40]. Navitoclax synergized with ado-trastuzumab emtansine (T-DM1) in HER2-positive patient-derived breast cancer xenograft models [45]. The phase 1 human trials showed that Navitoclax is safe and well tolerated, despite dose-dependent thrombocytopenia as a major adverse effect due to Bcl-xL inhibition [46]. Navitoclax is currently being tested in TNBC and ovarian cancer in a phase 1 trial in combination with Olaparib (NCT05358639). **Venetoclax/ABT-199**, a Bcl-2 selective inhibitor showed no benefit when tested in a randomized phase 2 study comparing fulvestant with or without venetoclax in patients with ER positive metastatic breast cancer after prior endocrine therapy combined with a CDK4/6 inhibitor [47]. A phase 1b study of venetoclax combined with tamoxifen in previously treated ER and Bcl-2 positive metastatic breast cancer was well tolerated and an overall response rate of 54% was observed [48]. Mcl-1 inhibitors have shown cytotoxic effect in breast cancer cell models [49-52]. **MIK665**, a Mcl-1 inhibitor, is currently being tested in clinical trials in hematologic malignancies, but not in breast cancers.

1.4. Other class of inhibitors

Doxycycline, a widely used antibiotic, is reported to inhibit mitochondrial biogenesis [53,54], and a phase 2 clinical trial in combination with metformin for patients with localized breast and uterine cancer is currently ongoing (NCT02874430). A recently developed, novel recombinant protein myc inhibitor, **Omomyc** (a.k.a., **OMO-103**), showed promising anti-tumor and anti-metastatic activities in breast cancer models both in vitro and in vivo [55], and currently is being tested in phase1/2 clinical trials with TNBC, non-small cell lung cancer and colorectal cancer (NCT04808362).

While there is much interest in targeting the mitochondria in breast cancer, the ongoing studies have not yet shown clear efficacy. Many challenges remain in mitochondria-targeted therapy such as the difficulty to develop specific inhibitors of metabolic enzymes due to multiple isoforms, the prevalence of hydrophobic pockets in these target enzymes, and the cancer cells’ ability to further reprogram metabolic pathways and survive [56,57].

Table S1. The status of development of mitochondria-targeting drugs in breast cancers (not including ClpP agonists).

Target	Drug	Mechanism of Action	Development stage in breast cancer (Phase)	Clinical trial	Target cancer types in clinical trial	Drug Combination	Status/ Results	Ref.
ETC	MitoTam	Complex I inhibitor	Clinical	EudraCT 2017-004441-25	unknown		ongoing (as of 2020)/ unknown	[9]
	α-TOS	Complex II inhibitor	Preclinical	N.A.				[58]
	MitoVES	Complex II inhibitor	Preclinical	N.A.				[58]
	IACS-010759	Complex I inhibitor	Preclinical	N.A.				[10]
	Metformin	Complex I inhibitor	Clinical (Ph2)	NCT04559308	locally advanced breast cancer, non-diabetic	chemotherapy	completed/no improvement	[21]
	Metformin	Complex I inhibitor	Clinical (Ph2)	NCT01310231	metastatic breast cancer, non-diabetic	chemotherapy	completed/no improvement	[22]
	Metformin	Complex I inhibitor	Clinical (Ph3)	NCT01101438	high-risk nonmetastatic breast cancer	chemotherapy	completed/no improvement	[23]
	ME-344	Complex I-V inhibitor	Clinical (Ph0/1)	NCT02806817	Early HER2-negative Breast Cancer	Bevacizumab	completed	[13]
	Leflunomide	DHODH inhibitor	Clinical (Ph1/2)	NCT03709446	previously treated metastatic TNBC		recruiting	[27]
TCA cycle	CPI-613 (Devimistat)	PDH and α-KGDH inhibitor	Preclinical		not in breast cancer			[28], [29]
glutamine metabolism	CB-839	Glutaminase inhibitor	Clinical (Ph2)	NCT03057600	TNBC	Paclitaxel	completed/ unknown	[32]
FAO	Etomoxir	CPT1 inhibitor	Preclinical	N.A.				[37], [38]

**Fatty acid synthesis**	TVB-2640	FASN inhibitor	Clinical (Ph2)	NCT03179904	HER2 Positive Metastatic Breast Cancer	Paclitaxel trastuzumab	recruiting	Phase1 study [33]
	bempedoic acid	ACLY inhibitor	Preclinical					[34]
	Omeprazole	FASN inhibitor/proton pump inhibitor	Clinical (Ph2)	NCT02595372	operable TNBC	prior to neoadjuvant anthracycline –taxane-based chemotherapy	completed	[36]
**Anti-apoptotic Bcl-2 family proteins**	Navitoclax (ABT-263)	Bcl-2/Bcl-xL inhibitor	Clinical (Ph1)	NCT05358639	Breast and Ovarian cancer	Olaparib	recruiting	[40]
	Venetoclax (ABT-199)	Bcl-2 inhibitor	Clinical (Ph2)	NCT03584009	ER-Positive Metastatic Breast Cancer Post-CDK4/6 Inhibitors	Fulvestrant	terminated/ do not indicate clinical utility	[47]
	Venetoclax (ABT-199)	Bcl-2 inhibitor	Clinical (Ph1/2)	NCT04298918	HER2 Positive Locally Advanced or Metastatic Breast Cancer	Trastuzumab Emtansine	terminated/ unknown	N.A.
	Venetoclax (ABT-199)	Bcl-2 inhibitor	Clinical (Ph1)	NCT04274933	ER+, HER2-Negative Locally Advanced or Metastatic Breast Cancer Who Had Disease Progression During or After CDK4/6 Inhibitor Therapy	Capecitabine	terminated/ unknown	N.A.
	Venetoclax (ABT-199)	Bcl-2 inhibitor	Clinical (Ph1)	NCT03900884	ER and BCL-2 positive locally advanced or metastatic breast cancer (PALVEN)	Palbociclib Letrozole	recruiting	[48]
	MIK665	Mcl-1 inhibitor	Preclinical	N.A.	Not in breast cancer			N.A.
**Translation**	Doxycycline	Mitochondrial biogenesis	Clinical (Ph2)	NCT02874430	localized breast and uterine cancer	Metformin	not recruiting/ unknown	N.A.

**Myc**	Omomyc/OMO-103	Myc inhibitor	Clinical (Ph1/2)	NCT04808362	TNBC and other solid tumors	not recruiting/ unknown	[55]

References

1. Huang, M.; Myers, C.R.; Wang, Y.; You, M. Mitochondria as a Novel Target for Cancer Chemoprevention: Emergence of Mitochondrial-targeting Agents. *Cancer Prev Res (Phila)* **2021**, *14*, 285-306, doi:10.1158/1940-6207.CAPR-20-0425.
2. Kafkova, A.; Trnka, J. Mitochondria-targeted compounds in the treatment of cancer. *Neoplasma* **2020**, *67*, 450-460, doi:10.4149/neo_2020_190725N671.
3. Dong, L.; Gopalan, V.; Holland, O.; Neuzil, J. Mitocans Revisited: Mitochondrial Targeting as Efficient Anti-Cancer Therapy. *Int J Mol Sci* **2020**, *21*, doi:10.3390/ijms21217941.
4. Sandoval-Acuña, C.; Fuentes-Retamal, S.; Guzmán-Rivera, D.; Peredo-Silva, L.; Madrid-Rojas, M.; Rebolledo, S.; Castro-Castillo, V.; Pavani, M.; Catalán, M.; Maya, J.D.; et al. Destabilization of mitochondrial functions as a target against breast cancer progression: Role of TPP(+)-linked-polyhydroxybenzoates. *Toxicol Appl Pharmacol* **2016**, *309*, 2-14, doi:10.1016/j.taap.2016.08.018.
5. De Francesco, E.M.; Ózsvári, B.; Sotgia, F.; Lisanti, M.P. Dodecyl-TPP Targets Mitochondria and Potently Eradicates Cancer Stem Cells (CSCs): Synergy With FDA-Approved Drugs and Natural Compounds (Vitamin C and Berberine). *Front Oncol* **2019**, *9*, 615, doi:10.3389/fonc.2019.00615.
6. Fuentes-Retamal, S.; Sandoval-Acuña, C.; Peredo-Silva, L.; Guzmán-Rivera, D.; Pavani, M.; Torrealba, N.; Truksa, J.; Castro-Castillo, V.; Catalán, M.; Kemmerling, U.; et al. Complex Mitochondrial Dysfunction Induced by TPP. *Cells* **2020**, *9*, doi:10.3390/cells9020407.
7. Gazzano, E.; Lazzarato, L.; Rolando, B.; Kopecka, J.; Guglielmo, S.; Costamagna, C.; Chegaev, K.; Riganti, C. Mitochondrial Delivery of Phenol Substructure Triggers Mitochondrial Depolarization and Apoptosis of Cancer Cells. *Front Pharmacol* **2018**, *9*, 580, doi:10.3389/fphar.2018.00580.
8. Ózsvári, B.; Sotgia, F.; Lisanti, M.P. First-in-class candidate therapeutics that target mitochondria and effectively prevent cancer cell metastasis: mitoriboscins and TPP compounds. *Aging (Albany NY)* **2020**, *12*, 10162-10179, doi:10.18632/aging.103336.
9. Rohlenova, K.; Sachaphibulkij, K.; Stursa, J.; Bezawork-Geleta, A.; Blecha, J.; Endaya, B.; Werner, L.; Cerny, J.; Zobalova, R.; Goodwin, J.; et al. Selective Disruption of Respiratory Supercomplexes as a New Strategy to Suppress Her2. *Antioxid Redox Signal* **2017**, *26*, 84-103, doi:10.1089/ars.2016.6677.
10. Evans, K.W.; Yuca, E.; Scott, S.S.; Zhao, M.; Paez Arango, N.; Cruz Pico, C.X.; Saridogan, T.; Shariati, M.; Class, C.A.; Bristow, C.A.; et al. Oxidative Phosphorylation Is a Metabolic Vulnerability in Chemotherapy-Resistant Triple-Negative Breast Cancer. *Cancer Res* **2021**, *81*, 5572-5581, doi:10.1158/0008-5472.CAN-20-3242.
11. Yap, T.A.; Daver, N.; Mahendra, M.; Zhang, J.; Kamiya-Matsuoka, C.; Meric-Bernstam, F.; Kantarjian, H.M.; Ravandi, F.; Collins, M.E.; Francesco, M.E.D.; et al. Complex I inhibitor of oxidative phosphorylation in advanced solid tumors and acute myeloid leukemia: phase I trials. *Nat Med* **2023**, doi:10.1038/s41591-022-02103-8.
12. Zhang, L.; Zhang, J.; Ye, Z.; Townsend, D.M.; Tew, K.D. Pharmacology of ME-344, a novel cytotoxic isoflavone. *Adv Cancer Res* **2019**, *142*, 187-207, doi:10.1016/bs.acr.2019.01.005.
13. Quintela-Fandino, M.; Morales, S.; Cortés-Salgado, A.; Manso, L.; Apala, J.V.; Muñoz, M.; Gasol Cudos, A.; Salla Fortuny, J.; Gion, M.; Lopez-Alonso, A.; et al. Randomized Phase 0/I Trial of the Mitochondrial Inhibitor ME-344 or Placebo Added to Bevacizumab in Early HER2-Negative Breast Cancer. *Clin Cancer Res* **2020**, *26*, 35-45, doi:10.1158/1078-0432.CCR-19-2023.
14. Owen, M.R.; Doran, E.; Halestrap, A.P. Evidence that metformin exerts its anti-diabetic effects through inhibition of complex 1 of the mitochondrial respiratory chain. *Biochem J* **2000**, *348 Pt 3*, 607-614.
15. Degli Esposti, M. Inhibitors of NADH-ubiquinone reductase: an overview. *Biochim Biophys Acta* **1998**, *1364*, 222-235, doi:10.1016/s0005-2728(98)00029-2.

16. Evans, J.M.; Donnelly, L.A.; Emslie-Smith, A.M.; Alessi, D.R.; Morris, A.D. Metformin and reduced risk of cancer in diabetic patients. *BMJ* **2005**, *330*, 1304-1305, doi:10.1136/bmj.38415.708634.F7.
17. Shi, P.; Liu, W.; Tala; Wang, H.; Li, F.; Zhang, H.; Wu, Y.; Kong, Y.; Zhou, Z.; Wang, C.; et al. Metformin suppresses triple-negative breast cancer stem cells by targeting KLF5 for degradation. *Cell Discov* **2017**, *3*, 17010, doi:10.1038/celldisc.2017.10.
18. Hirsch, H.A.; Iliopoulos, D.; Tsichlis, P.N.; Struhl, K. Metformin selectively targets cancer stem cells, and acts together with chemotherapy to block tumor growth and prolong remission. *Cancer Res* **2009**, *69*, 7507-7511, doi:10.1158/0008-5472.CAN-09-2994.
19. Liu, B.; Fan, Z.; Edgerton, S.M.; Deng, X.S.; Alimova, I.N.; Lind, S.E.; Thor, A.D. Metformin induces unique biological and molecular responses in triple negative breast cancer cells. *Cell Cycle* **2009**, *8*, 2031-2040, doi:10.4161/cc.8.13.8814.
20. Wang, J.C.; Li, G.Y.; Wang, B.; Han, S.X.; Sun, X.; Jiang, Y.N.; Shen, Y.W.; Zhou, C.; Feng, J.; Lu, S.Y.; et al. Metformin inhibits metastatic breast cancer progression and improves chemosensitivity by inducing vessel normalization via PDGF-B downregulation. *J Exp Clin Cancer Res* **2019**, *38*, 235, doi:10.1186/s13046-019-1211-2.
21. Barakat, H.E.; Hussein, R.R.S.; Elberry, A.A.; Zaki, M.A.; Ramadan, M.E. The impact of metformin use on the outcomes of locally advanced breast cancer patients receiving neoadjuvant chemotherapy: an open-labelled randomized controlled trial. *Sci Rep* **2022**, *12*, 7656, doi:10.1038/s41598-022-11138-3.
22. Pimentel, I.; Lohmann, A.E.; Ennis, M.; Dowling, R.J.O.; Cescon, D.; Elser, C.; Potvin, K.R.; Haq, R.; Hamm, C.; Chang, M.C.; et al. A phase II randomized clinical trial of the effect of metformin versus placebo on progression-free survival in women with metastatic breast cancer receiving standard chemotherapy. *Breast* **2019**, *48*, 17-23, doi:10.1016/j.breast.2019.08.003.
23. Goodwin, P.J.; Chen, B.E.; Gelmon, K.A.; Whelan, T.J.; Ennis, M.; Lemieux, J.; Ligibel, J.A.; Hershman, D.L.; Mayer, I.A.; Hobday, T.J.; et al. Effect of Metformin vs Placebo on Invasive Disease-Free Survival in Patients With Breast Cancer: The MA.32 Randomized Clinical Trial. *JAMA* **2022**, *327*, 1963-1973, doi:10.1001/jama.2022.6147.
24. Cejuela, M.; Martin-Castillo, B.; Menendez, J.A.; Pernas, S. Metformin and Breast Cancer: Where Are We Now? *Int J Mol Sci* **2022**, *23*, doi:10.3390/ijms23052705.
25. Fontaine, E. Metformin and respiratory chain complex I: the last piece of the puzzle? *Biochem J* **2014**, *463*, e3-5, doi:10.1042/BJ20141020.
26. Pernicova, I.; Korbonits, M. Metformin--mode of action and clinical implications for diabetes and cancer. *Nat Rev Endocrinol* **2014**, *10*, 143-156, doi:10.1038/nrendo.2013.256.
27. van der Heijden, J.W.; Oerlemans, R.; Tak, P.P.; Assaraf, Y.G.; Kraan, M.C.; Scheffer, G.L.; van der Laken, C.J.; Lems, W.F.; Scheper, R.J.; Dijkmans, B.A.; et al. Involvement of breast cancer resistance protein expression on rheumatoid arthritis synovial tissue macrophages in resistance to methotrexate and leflunomide. *Arthritis Rheum* **2009**, *60*, 669-677, doi:10.1002/art.24354.
28. Shen, N.; Korm, S.; Karantanos, T.; Li, D.; Zhang, X.; Ritou, E.; Xu, H.; Lam, A.; English, J.; Zong, W.X.; et al. DLST-dependence dictates metabolic heterogeneity in TCA-cycle usage among triple-negative breast cancer. *Commun Biol* **2021**, *4*, 1289, doi:10.1038/s42003-021-02805-8.
29. Córdova-Delgado, M.; Fuentes-Retamal, S.; Palominos, C.; López-Torres, C.; Guzmán-Rivera, D.; Ramírez-Rodríguez, O.; Araya-Maturana, R.; Urra, F.A. FRI-1 Is an Anti-Cancer Isoquinolinequinone That Inhibits the Mitochondrial Bioenergetics and Blocks Metabolic Shifts by Redox Disruption in Breast Cancer Cells. *Antioxidants (Basel)* **2021**, *10*, doi:10.3390/antiox10101618.
30. Alistar, A.; Morris, B.B.; Desnoyer, R.; Klepin, H.D.; Hosseinzadeh, K.; Clark, C.; Cameron, A.; Leyendecker, J.; D'Agostino, R., Jr.; Topaloglu, U.; et al. Safety and tolerability of the first-in-class agent CPI-613 in combination with modified

FOLFIRINOX in patients with metastatic pancreatic cancer: a single-centre, open-label, dose-escalation, phase 1 trial. *Lancet Oncol* **2017**, *18*, 770-778, doi:10.1016/S1470-2045(17)30314-5.

31. Philip, P.A.; Buyse, M.E.; Alistar, A.T.; Rocha Lima, C.M.; Luther, S.; Pardee, T.S.; Van Cutsem, E. A Phase III open-label trial to evaluate efficacy and safety of CPI-613 plus modified FOLFIRINOX (mFFX) versus FOLFIRINOX (FFX) in patients with metastatic adenocarcinoma of the pancreas. *Future Oncol* **2019**, *15*, 3189-3196, doi:10.2217/fon-2019-0209.
32. Gross, M.I.; Demo, S.D.; Dennison, J.B.; Chen, L.; Chernov-Rogan, T.; Goyal, B.; Janes, J.R.; Laidig, G.J.; Lewis, E.R.; Li, J.; et al. Antitumor activity of the glutaminase inhibitor CB-839 in triple-negative breast cancer. *Mol Cancer Ther* **2014**, *13*, 890-901, doi:10.1158/1535-7163.MCT-13-0870.
33. Falchook, G.; Infante, J.; Arkenau, H.T.; Patel, M.R.; Dean, E.; Borazanci, E.; Brenner, A.; Cook, N.; Lopez, J.; Pant, S.; et al. First-in-human study of the safety, pharmacokinetics, and pharmacodynamics of first-in-class fatty acid synthase inhibitor TVB-2640 alone and with a taxane in advanced tumors. *EClinicalMedicine* **2021**, *34*, 100797, doi:10.1016/j.eclinm.2021.100797.
34. Velez, B.C.; Petrella, C.P.; DiSalvo, K.H.; Cheng, K.; Kravtsov, R.; Krasniqi, D.; Krucher, N.A. Combined inhibition of ACLY and CDK4/6 reduces cancer cell growth and invasion. *Oncol Rep* **2023**, *49*, doi:10.3892/or.2022.8469.
35. Wang, C.J.; Li, D.; Danielson, J.A.; Zhang, E.H.; Dong, Z.; Miller, K.D.; Li, L.; Zhang, J.T.; Liu, J.Y. Proton pump inhibitors suppress DNA damage repair and sensitize treatment resistance in breast cancer by targeting fatty acid synthase. *Cancer Lett* **2021**, *509*, 1-12, doi:10.1016/j.canlet.2021.03.026.
36. Sardesai, S.D.; Thomas, A.; Gallagher, C.; Lynce, F.; Ottaviano, Y.L.; Ballinger, T.J.; Schneider, B.P.; Storniolo, A.M.; Bauchle, A.; Althouse, S.K.; et al. Inhibiting Fatty Acid Synthase with Omeprazole to Improve Efficacy of Neoadjuvant Chemotherapy in Patients with Operable TNBC. *Clin Cancer Res* **2021**, *27*, 5810-5817, doi:10.1158/1078-0432.CCR-21-0493.
37. Camarda, R.; Zhou, A.Y.; Kohnz, R.A.; Balakrishnan, S.; Mahieu, C.; Anderton, B.; Eyob, H.; Kajimura, S.; Tward, A.; Krings, G.; et al. Inhibition of fatty acid oxidation as a therapy for MYC-overexpressing triple-negative breast cancer. *Nat Med* **2016**, *22*, 427-432, doi:10.1038/nm.4055.
38. Wang, T.; Fahrmann, J.F.; Lee, H.; Li, Y.J.; Tripathi, S.C.; Yue, C.; Zhang, C.; Lifshitz, V.; Song, J.; Yuan, Y.; et al. JAK/STAT3-Regulated Fatty Acid β-Oxidation Is Critical for Breast Cancer Stem Cell Self-Renewal and Chemoresistance. *Cell Metab* **2018**, *27*, 1357, doi:10.1016/j.cmet.2018.04.018.
39. Lee, E.Y.; Gong, E.Y.; Shin, J.S.; Moon, J.H.; Shim, H.J.; Kim, S.M.; Lee, S.; Jeong, J.; Gong, J.H.; Kim, M.J.; et al. Human breast cancer cells display different sensitivities to ABT-263 based on the level of survivin. *Toxicol In Vitro* **2018**, *46*, 229-236, doi:10.1016/j.tiv.2017.09.023.
40. Lee, A.; Jin, H.O.; Masudul Haque, M.; Kim, H.Y.; Jung, H.; Park, J.H.; Kim, I.; Song, J.Y.; Yoon, H.K.; Kim, H.K.; et al. Synergism of a novel MCL-1 downregulator, acriflavine, with navitoclax (ABT-263) in triple-negative breast cancer, lung adenocarcinoma and glioblastoma multiforme. *Int J Oncol* **2022**, *60*, doi:10.3892/ijo.2021.5292.
41. Zoeller, J.J.; Vagodny, A.; Daniels, V.W.; Taneja, K.; Tan, B.Y.; DeRose, Y.S.; Fujita, M.; Welm, A.L.; Letai, A.; Leverson, J.D.; et al. Navitoclax enhances the effectiveness of EGFR-targeted antibody-drug conjugates in PDX models of EGFR-expressing triple-negative breast cancer. *Breast Cancer Res* **2020**, *22*, 132, doi:10.1186/s13058-020-01374-8.
42. Tiwari, S.; Kaur, H.; Anees, M.; Gupta, P.; Dalela, M.; Kharbanda, S.; Singh, H. Co-encapsulation of PI3-Kδ/HDAC6 dual inhibitor and Navitoclax in Quatramer™ nanoparticles for synergistic effect in ER+ breast cancer. *Int J Pharm* **2022**, *628*, 122343, doi:10.1016/j.ijpharm.2022.122343.
43. Vivo-Llorca, G.; Candela-Noguera, V.; Alfonso, M.; García-Fernández, A.; Orzáez, M.; Sancenón, F.; Martínez-Máñez, R. MUC1 Aptamer-Capped Mesoporous Silica Nanoparticles for Navitoclax Resistance Overcoming in Triple-Negative Breast Cancer. *Chemistry* **2020**, *26*, 16318-16327, doi:10.1002/chem.202001579.

44. Pesch, A.M.; Chandler, B.C.; Michmerhuizen, A.R.; Carter, H.M.; Hirsh, N.H.; Wilder-Romans, K.; Liu, M.; Ward, T.; Ritter, C.L.; Nino, C.A.; et al. Bcl-xL inhibition radiosensitizes. *Cancer Res Commun* **2022**, *2*, 679-693, doi:10.1158/2767-9764.crc-22-0024.
45. Zoeller, J.J.; Vagodny, A.; Taneja, K.; Tan, B.Y.; O'Brien, N.; Slamon, D.J.; Sampath, D.; Leverson, J.D.; Bronson, R.T.; Dillon, D.A.; et al. Neutralization of BCL-2/X. *Mol Cancer Ther* **2019**, *18*, 1115-1126, doi:10.1158/1535-7163.MCT-18-0743.
46. Gandhi, L.; Camidge, D.R.; Ribeiro de Oliveira, M.; Bonomi, P.; Gandara, D.; Khaira, D.; Hann, C.L.; McKeegan, E.M.; Litvinovich, E.; Hemken, P.M.; et al. Phase I study of Navitoclax (ABT-263), a novel Bcl-2 family inhibitor, in patients with small-cell lung cancer and other solid tumors. *J Clin Oncol* **2011**, *29*, 909-916, doi:10.1200/JCO.2010.31.6208.
47. Lindeman, G.J.; Fernando, T.M.; Bowen, R.; Jerzak, K.J.; Song, X.; Decker, T.; Boyle, F.; McCune, S.; Armstrong, A.; Shannon, C.; et al. VERONICA: Randomized Phase II Study of Fulvestrant and Venetoclax in ER-Positive Metastatic Breast Cancer Post-CDK4/6 Inhibitors - Efficacy, Safety, and Biomarker Results. *Clin Cancer Res* **2022**, *28*, 3256-3267, doi:10.1158/1078-0432.CCR-21-3811.
48. Muttiah, C.; Whittle, J.R.; Oakman, C.; Lindeman, G.J. PALVEN: phase Ib trial of palbociclib, letrozole and venetoclax in estrogen receptor- and BCL2-positive advanced breast cancer. *Future Oncol* **2022**, *18*, 1805-1816, doi:10.2217/fon-2021-1450.
49. Kotschy, A.; Szlavik, Z.; Murray, J.; Davidson, J.; Maragno, A.L.; Le Toumelin-Braizat, G.; Chanrion, M.; Kelly, G.L.; Gong, J.N.; Moujalled, D.M.; et al. The MCL1 inhibitor S63845 is tolerable and effective in diverse cancer models. *Nature* **2016**, *538*, 477-482, doi:10.1038/nature19830.
50. Merino, D.; Whittle, J.R.; Vaillant, F.; Serrano, A.; Gong, J.N.; Giner, G.; Maragno, A.L.; Chanrion, M.; Schneider, E.; Pal, B.; et al. Synergistic action of the MCL-1 inhibitor S63845 with current therapies in preclinical models of triple-negative and HER2-amplified breast cancer. *Sci Transl Med* **2017**, *9*, doi:10.1126/scitranslmed.aam7049.
51. Lee, T.; Christov, P.P.; Shaw, S.; Tarr, J.C.; Zhao, B.; Veerasamy, N.; Jeon, K.O.; Mills, J.J.; Bian, Z.; Sensintaffar, J.L.; et al. Discovery of Potent Myeloid Cell Leukemia-1 (Mcl-1) Inhibitors That Demonstrate in Vivo Activity in Mouse Xenograft Models of Human Cancer. *J Med Chem* **2019**, *62*, 3971-3988, doi:10.1021/acs.jmedchem.8b01991.
52. Campbell, K.J.; Mason, S.M.; Winder, M.L.; Willemsen, R.B.E.; Cloix, C.; Lawson, H.; Rooney, N.; Dhayade, S.; Sims, A.H.; Blyth, K.; et al. Breast cancer dependence on MCL-1 is due to its canonical anti-apoptotic function. *Cell Death Differ* **2021**, *28*, 2589-2600, doi:10.1038/s41418-021-00773-4.
53. Zhang, L.; Xu, L.; Zhang, F.; Vlashi, E. Doxycycline inhibits the cancer stem cell phenotype and epithelial-to-mesenchymal transition in breast cancer. *Cell Cycle* **2017**, *16*, 737-745, doi:10.1080/15384101.2016.1241929.
54. Ózsvári, B.; Magalhães, L.G.; Latimer, J.; Kangasmetsa, J.; Sotgia, F.; Lisanti, M.P. A Myristoyl Amide Derivative of Doxycycline Potently Targets Cancer Stem Cells (CSCs) and Prevents Spontaneous Metastasis, Without Retaining Antibiotic Activity. *Front Oncol* **2020**, *10*, 1528, doi:10.3389/fonc.2020.01528.
55. Massó-Vallés, D.e.a. MYC Inhibition Halts Metastatic Breast Cancer Progression by Blocking Growth, Invasion, and Seeding. **2022**, *2*, 110-130, doi:org/10.1158/2767-9764.CRC-21-0103.
56. Sainero-Alcolado, L.; Liaño-Pons, J.; Ruiz-Pérez, M.V.; Arsenian-Henriksson, M. Targeting mitochondrial metabolism for precision medicine in cancer. *Cell Death Differ* **2022**, *29*, 1304-1317, doi:10.1038/s41418-022-01022-y.
57. Stine, Z.E.; Schug, Z.T.; Salvino, J.M.; Dang, C.V. Targeting cancer metabolism in the era of precision oncology. *Nat Rev Drug Discov* **2022**, *21*, 141-162, doi:10.1038/s41573-021-00339-6.
58. Ghosh, P.; Vidal, C.; Dey, S.; Zhang, L. Mitochondria Targeting as an Effective Strategy for Cancer Therapy. *Int J Mol Sci* **2020**, *21*, doi:10.3390/ijms21093363.
